# Work-related musculoskeletal disorders among desludging operators in Uganda

**DOI:** 10.1186/s12891-024-07564-1

**Published:** 2024-06-13

**Authors:** Bridget Nagawa Tamale, Tonny Ssekamatte, John Bosco Isunju, Aisha Nalugya, Mujjabi Martin Mukasa, Arnold Tigaiza, Doreen Nakalembe, Winnifred K. Kansiime, Ceaser Kimbugwe, Jane Sembuche Mselle, Richard K. Mugambe

**Affiliations:** 1 Department of Disease Control and Environmental Health, Makerere University School of Public Health, Kampala, Uganda; 2 Department of Urban Water and Sewerage Services, Ministry of Water and Environment, Kampala, Uganda; 3 Programs Department, WaterAid Uganda, Kampala, Uganda

**Keywords:** Work-related Musculoskeletal disorders, Ergonomic hazards, Desludging operators, Uganda

## Abstract

**Background:**

Despite the limited evidence, desludging operators remain at a heightened risk of work-related musculoskeletal disorders (WMSDs). This study established the prevalence and predictors of WMSDs among desludging operators in Uganda.

**Methods:**

A digitalized structured questionnaire was used to collect cross-sectional data on musculoskeletal disorders and routine workplace activities from 303 desludging operators in 11 cities in Uganda. These cities were purposively selected based on the presence of a fecal sludge treatment plant or wastewater treatment plant. The Nordic Musculoskeletal Questionnaire (NMQ) was used to assess WMSDs. Simple random sampling with replacements was used to select respondents. Data were analyzed using STATA version 15.0. Modified Poisson Regression was used to measure the strength of association between the independent variables and WMSDs.

**Results:**

A total of 303 study participants were interviewed (97.7% response rate). The average age of the respondents was 34.0 years (SD ± 9.8). The prevalence of WMSDs among desludging operators was 29.7%. The body parts affected by MSDs were; the elbow for 4.6% (14/303), shoulder for 5.0% (15/303), and wrist/hand for 6.3% (19/303) of the respondents. At multivariable analysis, after controlling for age, desludging operators’ ability to influence the availability of equipment needed to do their work (APR = 0.45, 95% CI: 0.20–0.99), and feeling that everything done was an effort (APR = 1.70, 95% CI: 1.01–2.87) were significantly associated with WMSDs.

**Conclusion:**

The prevalence of WMSDs was high among desludging operators in Uganda. Desludging operators’ ability to influence the availability of equipment needed to do their work and frequency of feeling that everything done was an effort were significantly associated with WMSDs. Interventions should focus on ensuring adequate provision of ergonomic equipment and promoting practices that reduce the physical strain associated with desludging tasks. Additionally, comprehensive training programs addressing proper lifting techniques and posture awareness could significantly mitigate the risk of WMSDs among desludging-operators.

## Background

Globally, musculoskeletal disorders (MSDs) constitute a major occupational health problem [1]. Approximately, 1.71 billion people have MSDs worldwide [2]. MSDs are an underlying cause of death and disability and account for 6.0% of the total global disability-adjusted life years [3, 4]. The prevalence of MSDs in the African region ranges from 15% to 93.6% [5]. The back, neck, shoulders, and upper limbs account for more than 50% of global MSD cases [2, 6, 7]. Low back and neck pain were in 2017 the leading causes of years lived with disability (YLD) while MSDs in other body parts were also ranked in the top fifteen worldwide [8]. Over 20% of all work-related injuries and illnesses and about 25% of the annual workers’ compensation payments in Uganda are attributed to work-related musculoskeletal disorders (WMSDs), most notably back injuries [9].

WMSDs are primarily caused by the effects of the immediate working environment [10, 11] and include painful disorders of muscles, tendons, and nerves [12]. Examples of WMSDs include carpal tunnel syndrome, sprains, strains, and tears, tendonitis, back injury and back pain, arthritis, thoracic outlet syndrome, and tension neck syndrome [12, 13]. WMSDs significantly limit mobility and dexterity, leading to early retirement from work, and a low health-related quality of life [2]. Available evidence indicates that MSDs constitute 39% of all work-related health burdens on workers, which contributes to worker disability and absenteeism, low productivity, increase in sick leaves, compensation claims, and health care costs [14, 15].

The prevalence and effects of WMSDs although under-studied among various occupations [16, 17], are likely to be high among desludging operators, who in the current study, are defined as individuals responsible for the safe emptying, transportation, and disposal of faecal sludge from the septic tank/pits to desludging sites [18]. Desludging operations involve working in awkward positions, repetitive movements, working under extreme weather conditions, and manual handling of heavy loads and mechanical vibrations which escalate the risk of WMSDs. Besides, the high job demands, low job satisfaction, and work-related stress further aggravate the development of WMSDs [19–21]. These risk factors are aggravated by limited knowledge of occupational hazards, lack of control measures, and poorly designed workstations among others [22, 23].

Occupational health scientists and related institutions recommend the use of the hierarchy of controls to determine the most appropriate actions/measures for controlling workplace exposures. The hierarchy of controls includes (1) elimination, (2) substitution (replacement of the hazard), (3) engineering controls (isolating people from the hazard), (4) administrative controls, and 4) the use of personal protective equipment (PPE) [24–26]. Workplace measures aimed at reducing or eliminating WMSDs include ergonomic workplace redesign, changes in work methods, adjusting work schedules and workloads, job rotations, training, employee exercise and work hardening, provision and use of PPE, and medical management to reduce exposure [24–29]. However, the elimination of hazards is considered the most effective approach [26, 30].

To date, global institutions such as the United Nations agencies, governments, non-state actors, and employers have made concerted efforts to improve occupational health, safety, and well-being. At the global level, the International Labour Organisation (ILO) adopted more than 40 standards to guide the implementation of occupational safety and health (OSH) preventive and protective measures [31]. ILO, through the development of over 40 Codes of Practice, set out practical guidelines for public authorities, employers, workers, enterprises, and specialized occupational safety and health protection bodies to protect workers from occupation-related diseases and injuries [31]. In 2006, Uganda enacted the OSH Act, and its subsidiary legislation to improve the health and well-being of workers by making the registration of workplaces and equipment certification, inspection and monitoring of OSH activities, and training and sensitization mandatory [32, 33].

Despite the existence of international and local OSH standards and legislations, desludging operators still face enormous occupational risks leading to WMSDs [33]. Nonetheless, there is a dearth of evidence on the prevalence and predictors of WMSDs among desludging operators in low-resource settings including Uganda. This study, therefore, used MacDonald’s conceptual model on risk factors for the development of work-related musculoskeletal disorders to establish the prevalence and predictors of WMSDs among desludging operators in Uganda. The evidence generated by the current study could be used to inform the design of targeted interventions to prevent and manage WMSDs among desludging operators. This study’s findings may also be used to inform policy and practice in the field of occupational health and safety in Uganda and beyond.

## Materials and methods

### Study design, setting, and population

A cross-sectional study utilizing quantitative data collection methods was conducted among desludging operators in 11 cities in Uganda. These cities included Arua, Gulu, Soroti, Lira, Mbale, Jinja, Hoima, Masaka, Mbarara, Fort Portal, and the Greater Kampala Metropolitan Area (GKMA), which encompasses Kampala City and the adjacent districts of Wakiso and Mukono, and Mpigi [34]. Kampala, Uganda’s capital, and Masaka are located in the central region, while Fort Portal, Mbarara and Hoima are situated in the western region. Mbale, Jinja and Soroti cities are located in the eastern region [34], while Arua, Gulu and Lira are in the north [34]. Aside Kampala, all the study cities are encapsulated with the respective districts, whose total populations are provided in Table 1. These cities have a total of 14 wastewater or fecal sludge treatment plants (Table 1). In some cities, dumping bays are used by gulpers that come with thick sludge, while Fecal Sludge Treatment Plant (FSTP) are mainly used by cesspool trucks since these usually transport watery sludge. With the exception of Kampala that has a conventional treatment plant, other cities depend on lagoons. In cities where there are no dedicated facilities for receiving fecal sludge, cesspool trucks typically discharge their contents into a manhole located prior to the Grit Chamber. This dilutes the sludge with incoming sewage and enables the grit and solid waste to be separated and captured at the grit tank and screen, respectively.

**Table 1:** Population and treatment plants in the respective districts Uganda

No		City	District Population [35]	Type of treatment facility
1		Arua	389,500	Idofe WWTP
				Dadanum WWTP and FSTP
2		Gulu	225,500	Pece FSTP
3		Soroti	323,800	Soroto WWTP
4		Lira	262,300	Lira WWTP
				Apii Deconcentrated FSTP
5		Mbale	291,000	Mbale WWTP
6		Jinja	254,900	Jinja WWTP with a fecal sludge receiving bay
7		Hoima	277,800	Hoima WWTP
8		Masaka	237,200	Masaka WWTP
9		Mbarara	179,300	Mbarara WWTP
10		Fort Portal	103,800	Fort portal WWTP and FSTP
11		GKMA including Kampala City, Wakiso, Mukono and Mpigi districts)	6,043,300	Lubigi WWTP and FSTP
				Bugoloobi WWTP

*FSTP- Fecal Sludge Treatment Plant *WWTP-Wastewater Treatment Plant

### Sample size estimation

This paper utilised data of a larger study [36], which aimed at assessing the impact of using the sanitation safety planning tool on the occupational health and safety of de-sludging operators in Uganda. Based on the assumptions of a prevalence of MSDs among sanitation workers of 83.3% [37], a 5% margin of error, and a 95% confidence interval, we used the Kish Leslie formula for cross-sectional studies [38] to generate a sample of 214 participants. We also considered a design effect of 1.3 to cater for intra-cluster correlation and an increase in the sampling error [39], and a 10% non-response rate, which yielded a final sample of 310.

### Sampling

A total of 11 urban centres/ cities were purposively selected based on the presence of FSTP or WWTP. Given that all the cities had a FSTP or WWTP, they were included in the sampling frame. Kampala City and its neigbourhood (Wakiso and Mukono districts) were considered as one urban centre (GKMA) given the fact that they shared FSTP or WWTP, and that the offices of the desludging companies were spread within the region. Thereafter, the research team contacted the Urban Water Supply and Sewerage Services Department of the Ministry of Water and Environment, the respective City Health Offices (Environmental Health departments) and the Private Emptiers Association of Uganda (an umbrella body for desludging operators) to obtain a list of companies engaged in desludging operations. These companies were later contacted for administrative clearance, and a list of the desludging operators they employed during the time of the survey. The Microsoft Excel computer-generator was then used to randomly select study participants.

### Conceptual framework and study variables

#### Conceptual model

**Fig. 1:**
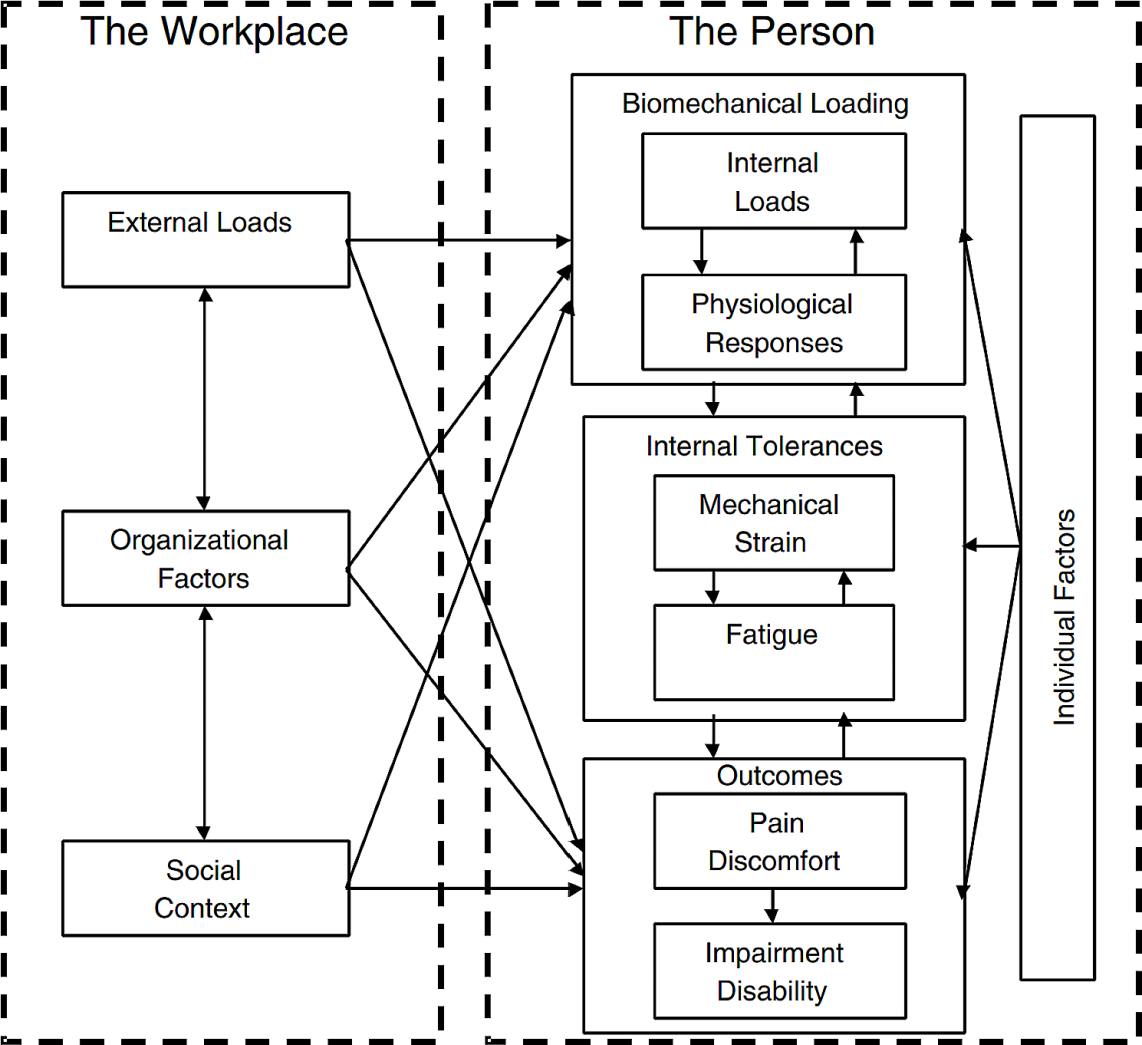
A conceptual model on risk factors for the development of work-related musculoskeletal disorders [40, 43]

The current study adapted variables in the conceptual framework for development of a toolkit for prevention of work-related musculoskeletal disorders [40]. The model postulates that WMSDs develop as a consequence of hazardous jobs and task demands, psychological demands, and a lack of effective coping strategies [40]. A multitude of hazards comprising of external loads, organisational factors and the social context elevate a worker’s risk of development of MSDs. The interaction of these hazards affects the internal biochemical processes and physiological responses such as stress, depression and burnout [41, 42], thus contributing to the development of MSD indicators such as discomfort, numbness, pain and injury [40, 43] (Fig. 1).

#### Outcome variable

A work-related musculoskeletal disorder was defined as injuries and disorders that affect the human body’s movement or musculoskeletal system (i.e. muscles, tendons, ligaments, nerves, discs, blood vessels, etc.) [44], resulting from desludging activities. Examples of MSDs included sprains, strains, tears and backpain [44]. The study adopted the Nordic Musculoskeletal Questionnaire (NMQ), an inexpensive instrument that is widely used to collect data on WMSDs [45–47]. The NMQ is used to collect data on the history of pain, discomfort, numbness or ache in the last 12 months, and whether the outcome (pain, discomfort, numbness or ache) prevented the respondent from effectively engaging in desludging activities. The prevalence of WMSDs was established by asking respondents if they had developed work-related musculoskeletal disorders in the last 12 months. This was coded as “1” for respondents who reported pain, discomfort, numbness or ache in any of the body parts and “0” for those who did not. Subjective ache/pain/numbness/injury was measured for the different body parts including the neck, shoulder, lower back, upper back, hips/thighs, knees, ankles/feet, and head.

#### Independent variables

Our independent variables included respondent background characteristics (such as age, sex, and level of education), work experience, working hours per week, weekly rest, terms of employment, self-perceived social status, receiving safety training and health-related educational sessions, and undertaking regular health check-up. Continuous data on age were later categorized at analysis as “18–30 years”, “31–43 years” and “44 years and above.” Duration of work per week was categorized as “48 hours and below” and “above 48 hours.” Education level was collected as a categorical variable grouped into “no formal education”, “primary”, “secondary” and tertiary, and was categorized at analysis into “primary and below”, for those who had never attended school or had only attained primary education; and “secondary and above” for respondents who had only attained secondary education or above secondary level. Duration of work experience was categorized into “5 years and below”, and “above 5 years”. The adult version of the MacArthur scale of subjective social status (SSS) was used to assess the respondents perceived social rank relative to other community members [48, 49]. We adopted the classification of subjective social status (SSS) used by Chen, Covinsky [50]. Thus, continuous data generated by asking the respondents’ SSS were further categorized as low (0–3), middle (4–7), and high (8–10).

Other independent variables included ergonomic characteristics and psychosocial risk factors. Ergonomic risk factors were assessed by collecting data on the following; postures adopted at work, heavy or frequent lifting/lowering/shoveling, hand force, repetitive work, and vibrating tools, bouncing or jarring and static postures and pushing and pulling against an object with maximum effort. Responses to each of the questions were categorized using an appropriate Likert scale. The psychosocial risk factors were measured using a modified version of the upper limb core QX checklist [51]. We assessed the following domains; job demand (five questions), job satisfaction and security (six questions), job control (four questions), work relationship (four questions), and mental state (five questions). For job demand, satisfaction, and security, respondents rated their responses on a Likert scale (Strongly disagree; Disagree; Neutral; Agree and Strongly agree). At analysis, these responses were merged into three categories (agree, neutral and disagree). For job control, respondents rated their responses using “very little”, “little”, moderate”, “much” and “very much”. These were each categorized into “little” “moderate” and “much” at analysis. Regarding work relationship, questions were asked about receiving support from supervisors and co-workers. Respondents rated this on a Likert scale from “very much/very easy”, “much/easy”, “a little” and “not at all”. This was later categorized as “very much”, “little” and “not at all.” Lastly respondents rated their mental state during the past month using these responses; “rarely or none of the time”, “sometimes”, “often” and “most or all of the time.” The questions were later categorized into “rarely or none of the time”, “sometimes” and “often.”

### Data collection, management and analysis

Face-to-face interviews were conducted using an electronic structured questionnaire designed using the Kobo Collect server, and pre-installed on smart mobile devices. The questionnaire was used to obtain data on the individual characteristics (such as age, sex, education level, marital status, work experience, number of hours worked per week, attendance of training and health-related education sessions, undertaking a regular medical checkup), history of WMSDs, and ergonomic and psychosocial risk factors. The data collection tool was developed after a thorough review of literature [52–54]. It was also validated by a team of occupational health specialists based at the Makerere University School of Public Health. On completion of data collection, data were downloaded from the Kobo Collect server (https://www.kobotoolbox.org/) and exported to Ms. Excel for cleaning. In order to control for response bias, we used neutral question wording, assured participants of anonymity and data confidentiality. Also, the use of relatively short recall period of 12 months and the use of visual aids in the questionnaire helped mitigate recall bias. Data cleaning involved the identification of duplicate data, errors, outliers, and inconsistencies, and rectifying them. Data were then exported to STATA version 15 for analysis. Continuous data were expressed as mean and standard deviation whereas categorical data were reported as frequencies and proportions. Modified Poisson Regression was used to measure the strength of association between the independent variables and WMSDs. At bivariate analysis, variables with a *p*-value ≤ 0.2 were included in the final model. Only variables with a *p*-value ≤ 0.05 were considered to be statistically significant at multivariable analysis.

### Quality control and assurance

Research assistants with a minimum of a bachelor’s degree were recruited and they underwent a 7-days training on the study protocol and ethical issues about the study. The data collection tool was pretested and translated into the local dialects. The electronic questionnaire was designed with skip patterns and validation mechanisms to ensure quality collection. Research assistants were supervised by supervisors, who were in return accountable to the core research team. Daily debrief meetings were held with the research assistants to identify any challenges that arose during the data collection process.

## Results

### Background characteristics of study participants

A total of 303 study participants were interviewed (97.7% response rate). Of these, 43.2% (131/303) were aged between 18 and 30 years. The average age of the respondents was 34.0 years (SD ± 9.8). Almost all, 97.0 (294/303) of the respondents were male, and 64.0% (194/303) had a secondary and above education. Over half, 54.1% (164/303) of the respondents had a work experience of 5 years and less, 56.4% (171/303) worked less than 48 h per week and majority, 62.0% (188/303) had at least a day rest per week. Majority, 90.8% (275/303) of the respondents had no formal employment contract, more than two thirds, 71.6% (217/303) were engaged in mechanical transport followed by 64.7% (196/303) were involved in mechanical emptying, 64.7% (196/303) however, only, 8.2% (25/303) of the respondents were engaged in the treatment process. Two thirds, 66.0% (200/303) of the respondents reported having a middle SSS, more than three quarters, 80.2% (243/303) had never attended safety training in the last 12 months and 54.1% (164/303) did not receive health-related educational sessions or orientation before being employed (Table 2).

**Table 2:** Background characteristics of participants

Variable	Frequency (*N* = 303)	Percentage (%)
**Age of respondent**		
18–30	131	43.2
31–43	117	38.6
≥ 44	55	18.2
**Sex of the respondent**		
Female	9	3.0
Male	294	97.0
**Highest level of formal education**		
Primary and below	109	36.0
Secondary and above	194	64.0
**Religious Affiliation**		
Christian	205	67.7
Muslim	98	32.3
**Desludging operator’s household size**		
1–5	185	61.1
6–10	90	29.7
Above 10	28	9.2
**Work experience (years)**		
≤ 5	164	54.1
> 5	139	45.9
**Working hours per week**		
≤ 48 h	171	56.4
> 48 h	132	43.6
**Weekly rest**		
No rest	115	37.9
1 and more days	188	62.0
**Formal employment contract**		
No	275	90.8
Yes	28	9.2
**Nature of contract (** *n* ** = 28)**		
Full time and permanent	14	50.0
Full time and temporary	5	17.9
Part time and permanent	4	14.3
Part time and temporary	5	17.9
**If No contract, nature of engagement**		
Full time and permanent	103	37.4
Full time and temporary	91	33.1
Part time and permanent	14	5.1
Part time and temporary	67	24.4
Stage of sanitation chain engaged in *		
Emptying	250	82.5
Conveyance/ transportation	233	76.9
Treatment	25	8.2
End-use or disposal	229	75.6
**Social status**		
Low SSS	103	34.0
Middle SSS	200	66.0
**Attended safety training in the last 12 months**		
No	243	80.2
Yes	60	19.8
**Attended safety training in the last 6 months**		
No	274	90.4
Yes	29	9.6
**Received health-related educational sessions or orientation before being employed**		
No	164	54.1
Yes	139	45.9
**Undertaking regular health check-up**		
No	243	80.2
Yes	60	19.8

Multiple response *

### Prevalence of work-related musculoskeletal disorders

**Fig. 2:**
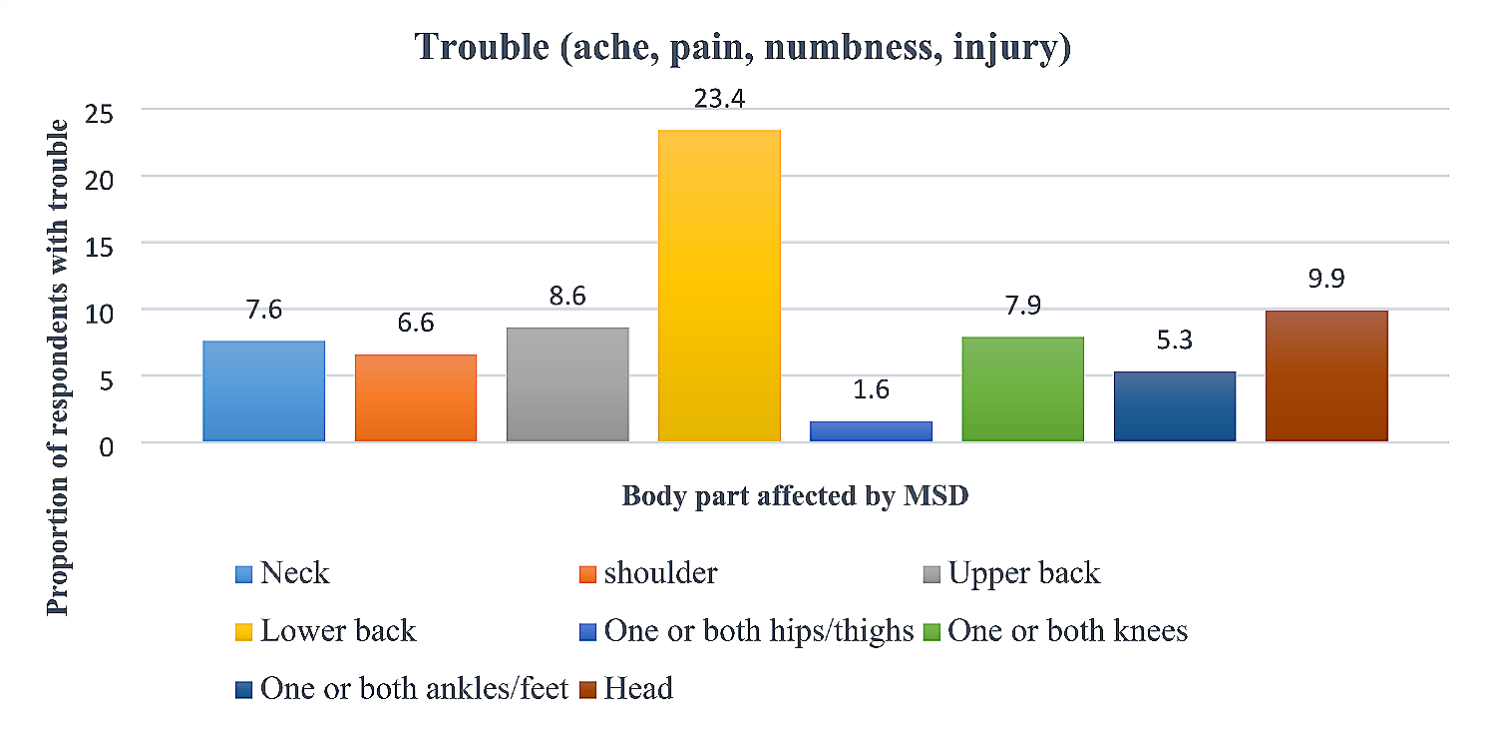
Body parts mainly affected by MSDs among desludging operators in Uganda

Nearly half, 49.8% (151/303) of the respondents reported having had a MSD within the last 12 months. About 29.7% (90/303) reported that the MSD experienced in the last 12 months was work-related (WMSD). Only 63.3% (57/90) of the respondents who had experienced a WMSD reported it to their employers. More than a third, 34.0% (103/303) of the respondents reported having been prevented from doing normal work (at home or away from home) because of a WMSD in the last 12 months. The body parts affected by MSDs were; the elbow for 4.6% (14/303), shoulder for 5.0% (15/303), and wrist/hand for 6.3% (19/303) of the respondents (Fig. 2).

### Ergonomic characteristics of the study participants

More than half, 59.7% (181/303) of the respondents reported having never lifted or lowered objects more than 50 kg per day, 58.4% (177/303) occasionally lifted or lowered objects more than 25 kg per day whereas 62.4% (189/303) occasionally shoveled per day. Additionally, about 18.8% (57/303) never worked with their hand above the head per day, 69.3% (210/303) occasionally worked with their neck bent more than 30 degrees without support, 69.0% (209/303) occasionally worked with a bent wrist, 7.6% (23/303) never worked with back bent without support, 2.0% (6/303) worked more than 4 h per day while squatting, and 49.5% (150/303) occasionally worked while kneeling.

Majority, 53.1% (161/303) of the respondents never pinched unsupported objects, 47.2% (143/303) occasionally grasped an unsupported object weighing 5 or more kg per hand, or grasped with a forceful, and 57.4% (174/303) occasionally grasped object with the wrist bent per day. About, 39.9% (121/303) of the respondents occasionally repeated the same motion with little or no variation every few seconds, 68.0% (206/303) never used grinders, jig saws or other hand tools that typically have moderate vibration levels, and 71.6% (217/303) never used impact wrenches, chain saws, percussive tools (jackhammers, scalers, chipping hammers) or other tools that typically have high vibration levels.

A few, 29.4% (89/303) of the respondents occasionally operated mobile equipment per day, and more than a half 52.2% (106/203) of the respondents who operated mobile equipment most of the time traveled over rough roads. Close to quarter, 42.9% (130/303) occasionally stood without changing position per day while 39.6% (120/303) occasionally sat without changing position. More than a third, 38.6% (117/303) of the respondents occasionally pushed against an object such as a wheelbarrow with a maximum effort per day, and 40.6% (123/303) occasionally pushed against an object such as a wheelbarrow with a moderate effort. More than half, 50.2% (152/303) of the respondents never pulled against an object, like an electric cable, fuel hose, or wheelbarrow with a maximum effort while 51.2% (155/303) never pulled against an object, like a/an electric cable, fuel hose or wheelbarrow with a moderate effort (Table 3).

**Table 3:** Description of ergonomic indicators among desludging operators in Uganda

Ergonomic Factors	Attribute	Frequency (*n* = 303)	Percentage (%)
**Heavy or Frequent lifting/lowering/shoveling**			
Lift or lower objects more than 50 kg per day	Greater or equal to 10 times/day	4	1.3
	Less than 10 times/day	17	5.6
	Never	181	59.7
	Occasionally	101	33.3
Shoveling per day	2–4 h per day	12	4.0
	Less than 2 h per day	57	18.8
	More than 4 h per day	8	2.6
	Never	37	12.2
	Occasionally	189	62.4
**Awkward Postures**			
Hand above the head	2–4 h per day	8	2.6
	Less than 2 h per day	30	9.9
	More than 4 h per day	4	1.3
	Never	57	18.8
	Occasionally	204	67.3
Squatting	2–4 h per day	8	2.6
	Less than 2 h per day	40	13.2
	More than 4 h per day	6	2.0
	Never	64	21.1
	Occasionally	185	61.1
Kneeling	2–4 h per day	6	2.0
	Less than 2 h per day	23	7.6
	More than 4 h per day	1	0.3
	Never	123	40.6
	Occasionally	150	49.5
**High hand force- Pinch and power grip**			
Pinch unsupported objects	2–4 h per day	1	0.3
	Less than 2 h per day	10	3.3
	More than 4 h per day	2	0.7
	Never	161	53.1
	Occasionally	129	42.6
Grasping object with wrist bent	2–4 h per day	7	2.3
	Less than 2 h per day	37	12.2
	More than 4 h per day	11	3.6
	Never	74	24.4
	Occasionally	174	57.4
**Highly repetitive work and vibrating tools (hand arm vibration**			
Repeating same motion with little or no variation every few seconds	2–4 h per day	15	5.0
	Less than 2 h per day	13	4.3
	More than 4 h per day	19	6.3
	Never	135	44.6
	Occasionally	121	39.9
**Bouncing or jarring (whole body vibrations) and static postures**			
Operating mobile equipment	2–4 h per day	52	17.2
	Less than 2 h per day	29	9.6
	More than 4 h per day	33	10.9
	Never	100	33.0
	Occasionally	89	29.4
Travel over rough roads (*n* = 203)	All the time	35	17.2
	Most of the time	106	52.2
	Never	1	0.5
	Sometimes	61	30.0
Sitting without changing position	2–4 h per day	35	11.6
	Less than 2 h per day	46	15.2
	More than 4 h per day	17	5.6
	Never	85	28.1
	Occasionally	120	39.6
**Pushing and pulling**			
Pushing against an object such as a wheelbarrow with a maximum effort	8–30 times per day	1	0.3
	Less than 8 times/day	14	4.6
	Never	171	56.4
	Occasionally	117	38.6
Pulling against an object, like a/an electric cable, fuel hose or wheelbarrow with a moderate effort	16–50 times	2	0.7
	Less than 16 times	14	4.6
	Never	155	51.2
	Occasionally	132	43.6

### Psychosocial characteristics of participants

Almost two thirds, 61.4% (186/303) of the respondents agreed that their job requires working very fast, 68.3% (207/303) agreed that their job requires working very hard, and 48.2% (146/303) agreed that they are NOT asked to do an excessive amount of work. More than half, 60.1% (182/303) agreed that they have enough time to get the job done, 47.9% (145/303) agreed that their job requires that they learn new things, 42.9% (130/303) disagreed that they can influence the availability of equipment needed to do their work, and more than a third, 42.9% (130/303) agreed that they can take a break when they want to. More than three quarters, 77.9% (236/303) of the respondents agreed that their supervisor is willing to listen to work-related problems, 58.1% (176/300) agreed that they have job security, and a half, 89.8% (272/303) agreed that their job requires a great deal of concentration.

Majority, 59.4% (180/303) agreed that they experience constant pressure from workgroup to keep up, 75.6% (229/303) agreed that their employer cares about health and safety on the job, and 61.4% (186/303) agreed that they receive the training needed to do my job well. Majority, 44.9% (136/303) of the respondents reported having much influence over the variety of tasks performed, 38.0% (115/303) reported having moderate influence over the amount of work done, 39.9% (121/303) reported to have much influence over the pace of work, that is how fast or slow they work, and 38.6% (117/303) reported to have much influence over the hours they work.

About a third, 56.8% (172/303) reported having much support from a supervisor when things get tough at work, 53.8% (163/303) reported that it is easy to talk with their immediate supervisor/boss, 57.4% (174/303) reported that their co-workers can be easily relied upon when things get tough at work, and 54.1% (164/303) reported that it is easy to talk with co-workers. Majority, 60.7% (184/303) of the respondents reported that they sometimes felt that everything done was an effort, 64.4% (195/303) reported that they felt happy, 48.2% (146/303) reported that they rarely felt depressed, 4.3% (13/303) reported that they often felt that people were unfriendly, and over half, 50.2% (152/303) reported that they rarely felt nervous (Table 4).

**Table 4:** Psychosocial characteristics of desludging operators in Uganda

Variable	Attribute	Frequency (*N* = 303)	Percentage (%)
**Job Demand**			
Job requires working very fast	Disagree	72	23.8
	Neutral	45	14.9
	Agree	186	61.4
Job requires working very hard	Disagree	38	12.5
	Neutral	58	19.1
	Agree	207	68.3
Have enough time to get the job done	Disagree	43	14.2
	Neutral	78	25.7
	Agree	182	60.1
Job requires that I learn new things	Disagree	82	27.1
	Neutral	76	25.1
	Agree	145	47.9
Able to influence the availability of equipment needed to do my work.	Disagree	130	42.9
	Neutral	65	21.5
	Agree	108	35.6
**Job satisfaction and security**			
Supervisor willing to listen to work-related problems	Agree	236	77.9
	Disagree	67	22.1
Have job security	Agree	176	58.1
	Disagree	127	41.9
Employer cares about health and safety on the job	Agree	229	75.6
	Disagree	74	24.4
Receive the training needed to do my job well	Agree	186	61.4
	Disagree	117	38.6
**Job control**			
Influence over the variety of tasks performed	Little	90	29.7
	Moderate	77	25.4
	Much	136	44.9
Influence over the amount of work done	Little	86	28.4
	Moderate	102	33.7
	Much	115	38.0
Influence over the pace of your work, that is how fast or slow you work	Little	82	27.1
	Moderate	100	33.0
	Much	121	39.9
Influence over the hours that you work	Little	95	31.4
	Moderate	91	30.0
	Much	117	38.6
**Work relationship**			
Supervisor can be relied upon when things get tough at work	Much/easy	172	56.8
	A little	104	34.3
	Not at all	27	8.9
Co-workers being relied upon when things get tough at work	Much/easy	174	57.4
	A little	102	33.7
	Not at all	27	8.9
**Mental state**			
Frequency of feeling that everything done was an effort	Rarely or none of the time	71	23.4
	Sometimes	184	60.7
	Often	48	15.8
Frequency of feeling happy	Rarely or none of the time	41	13.5
	Sometimes	195	64.4
	Often	67	22.1
Frequency of feeling depressed	Rarely or none of the time	146	48.2
	Sometimes	132	43.6
	Often	25	8.3
Frequency of feeling nervous	Rarely or none of the time	152	50.2
	Sometimes	129	42.6
	Often	22	7.3

### Predictors of work-related musculoskeletal disorders among desludging operators in Uganda

In multivariable regression, after controlling for age, ability to influence the availability of equipment needed to do their work, and frequency of feeling that everything done was an effort were significantly associated with WMSDs. Respondents who neither agreed nor disagreed about being able to influence the availability of equipment needed to do their work had a 55% lower prevalence of WMSDs as compared to those who disagreed (APR = 0.45, 95% CI: (0.20–0.99). Respondents who sometimes felt that everything done was an effort had a 70% higher prevalence of WMSDs as compared to their counterpart (APR = 1.70, 95% CI: 1.01–2.87) (Table 5).

**Table 5:** Predictors of work-related musculoskeletal disorders among desludging operators in Uganda

Background characteristics	WMSDs in the past 12 months		Crude PR (95% CI)	*P*-values	Adjusted PR (95% CI)	*P*-values
	Yes (*n* = 90)	No (*n* = 213)				
	F (%)	F (%)				
**Age**						
18–30	35 (38.9)	96 (45.1)	1		1	
31–43	36 (40.0)	81 (38.0)	1.15 (0.78–1.71)	0.482	1.16 (0.78–1.73)	0.457
≥ 44	19 (21.1)	36 (16.9)	1.29 (0.81–2.05)	0.276	1.14 (0.70–1.84)	0.596
**Job Demands**						
**Job requires working very fast**						
Disagree	15 (16.7)	57 (26.8)	1		1	
Neutral	11 (12.2)	34 (16.0)	1.17 (0.59–2.32)	0.647	1.31 (0.61–2.77)	0.486
Agree	64 (71.1)	122 (57.3)	1.65 (1.01–2.70)	**0.046**	1.43 (0.76–2.68)	0.262
**Job requires working very hard**						
Disagree	7 (7.8)	31 (14.6)	1		1	
Neutral	14 (15.6)	44 (20.7)	1.31 (0.58–2.95)	0.514	1.47 (0.58–3.79)	0.415
Agree	69 (76.7)	138 (64.8)	1.81 (0.90–3.63)	**0.096**	1.30 (0.58–2.90)	0.526
**Job requires that I learn new things**						
Disagree	31 (34.4)	51 (23.9)	1		1	
Neutral	21 (23.3)	55 (25.8)	0.73 (0.46–1.15)	**0.180**	1.00 (0.58–1.73)	0.993
Agree	38 (42.2)	107 (50.2)	0.69 (0.47–1.02)	**0.066**	0.70 (0.43–1.14)	0.155
**Able to influence the availability of equipment needed to do their work**						
Disagree	46 (51.1)	84 (39.4)	1		1	
Neutral	11 (12.2)	54 (25.4)	0.48 (0.26–0.86)	**0.014**	0.45 (0.20–0.99)	**0.048**
Agree	33 (36.7)	75 (35.2)	0.86 (0.60–1.25)	0.434	0.79 (0.51–1.22)	0.290
***Job Satisfaction and Security***						
**In their job, there is constant pressure from my work group to keep up**						
Agree	47 (52.2)	133 (62.4)	1		1	
Disagree	43 (47.8)	80 (37.6)	1.34 (0.95–1.89)	**0.097**	1.05 (0.67–1.67)	0.820
**Employer cares about their health and safety on the job**						
Agree	61 (67.8)	168 (78.9)	1		1	
Disagree	29 (32.2)	45 (21.1)	1.47 (1.03–2.10)	**0.034**	1.21 (0.84–1.75)	0.299
***Job Control***						
**Influence over the amount of work done**						
Little	22 (24.4)	64 (30.0)	1		1	
Moderate	25 (27.8)	77 (36.2)	0.96 (0.58–1.57)	0.866	1.21 (0.69–2.11)	0.497
Much	43 (47.8)	72 (33.8)	1.46 (0.95–2.25)	**0.085**	1.55 (0.91–2.62)	0.105
***Work relationship***						
**Supervisor can be relied upon when things get tough at work**						
Much/easy	49 (54.4)	123 (57.7)	1		1	
A little	28 (31.1)	76 (35.7)	0.94 (0.64–1.40)	0.780	1.07 (0.68–1.67)	0.780
Not at all	13 (14.4)	14 (6.6)	1.69 (1.07–2.67)	**0.025**	1.33 (0.76–2.32)	0.309
***Mental state***						
**Frequency of feeling that everything done was an effort**						
Rarely or none of the time	14 (15.6)	57 (26.8)	1		1	
Sometimes	59 (65.6)	125 (58.7)	1.63 (0.97–2.72)	**0.064**	1.70 (1.01–2.87)	**0.046**
Often	17 (18.9)	31 (14.6)	1.80 (0.98–3.29)	**0.058**	1.60 (0.85–3.02)	0.148
***Awkward Postures***						
**Bent wrist**						
2–4 h per day	3 (3.3)	2 (0.9)	1		1	
Less than 2 h per day	12 (13.3)	16 (7.5)	0.71 (0.31–1.65)	0.430	0.75 (0.25–2.28)	0.617
More than 4 h per day	2 (2.2)	9 (4.2)	0.30 (0.07–1.29)	**0.106**	0.34 (0.06–1.89)	0.217
Never	9 (10.0)	41 (19.2)	0.30 (0.12–0.76)	**0.011**	0.37 (0.11–1.20)	0.099
Occasionally	64 (71.1)	145 (68.1)	0.51 (0.24–1.07)	**0.077**	0.71 (0.25–2.05)	0.532
**Shoveling per day**						
2–4 h per day	2 (2.2)	10 (4.7)	1		1	
Less than 2 h per day	16 (17.8)	41 (19.2)	1.68 (0.44–6.39)	0.444	1.79 (0.50–6.39)	0.370
More than 4 h per day	2 (2.2)	6 (2.8)	1.50 (0.26–8.60)	0.649	1.70 (0.28–10.48)	0.566
Never	17 (18.9)	20 (9.4)	2.76 (0.74–10.26)	**0.131**	2.73 (0.77–9.71)	0.121
Occasionally	53 (58.9)	136 (63.8)	1.68 (0.46–6.10)	0.428	1.82 (0.54–6.17)	0.337

## Discussion

This study assessed the prevalence and predictors of WMSDs and thus providing a benchmark profile for desludging operators in low-resource settings. A relatively high prevalence of WMSDs among desludging operators was established possibly due to the nature of desludging activities [55]. Desludging operations are associated with changes in posture, rapid and abrupt flexion movements, and use of excessive force in the upper extremities, particularly the arms and hands, lower back, and so on [55]. Also, the adoption of a posture can occasionally be irreversible. The aforementioned workers spend a lot of time working in poor postures, using brooms and mops, hauling large objects, pushing carts for moving rubbish, among others. This exposes them to WMSDs which compromises their productivity, and health and could even cause disability and mortality. Work associated with repetitive motions and adopting different postures as was the case for the desludging operators, creates a risk for WMSDs [56]. The prevalence in this study was lower than the global prevalence of WMSDs among sanitation workers in low income countries [57]. Additionally, studies in other low-and-middle income countries showed a much-higher prevalence of WMSDs with over (90.8%) in India [58], 92.5% in Shiraz [59], 88.2% in Brazil and Rio Grande [60], and 61.3% in Nigeria port [61]. The body parts susceptible to WMSDs in our study did not differ from those reported in other studies, which indicated that the lower back, as well as the head, upper back, and other body parts, were the most commonly affected [47, 59]. The lower back was the most affected body part in this study, which is consistent with previous studies that have identified low-back pain as the most common WMSDs complaint ever reported [47, 59, 62, 63].

The factors associated with WMSDs among desludging operators in this study were: desludging operators’ ability to influence the availability of needed equipment, and frequency of feeling that everything done was an effort. This study noted that desludging operators’ ability to influence the availability of equipment needed to do their work was significantly associated with WMSDs. The relationship between WMSDs and the availability of equipment is critical [57, 64], and such a lack of necessary tools or using improper equipment for desludging tasks leads to strain or poor ergonomics. When desludging operators have the ability to choose or influence the tools they use, they are likely to select equipment that minimizes physical strain and meets their specific job demands, leading to reduced ergonomic risks. Psychologically, having control over one’s work environment, such as equipment choice, enhances job satisfaction, reduces stress, and indirectly lowers the risk of WMSDs by decreasing stress-related muscle tension [65]. Moreover, being involved in equipment decisions may also correlate with better training and awareness of safe work practices, further preventing injuries. Therefore, reducing WMSDs can result in a decrease in healthcare costs, an improvement in worker productivity and welfare, and a potential model for occupational health improvements that could be adapted across various industries. Addressing these issues not only improves the immediate working conditions of desludging operators but also offers insights into policy changes that could enhance occupational health standards, emphasizing the critical role of worker involvement in safety and equipment decisions. Our study findings are consistent with a study conducted by Tolera, Diriba [64], which denoted that lifting heavy loads without proper lifting aids or using inadequate protective gear might contribute to WMSDs.

Our study found a significant association between desludging operators who sometimes felt like everything they did was an effort and their risk of WMSDs. This could be explained by a psychological phenomenon where constant strain and frustration with the job, potentially due to factors like inadequate equipment or long hours, manifests as a sense of overall effort in daily tasks. This mental fatigue can lead to decreased focus and impaired judgement, increasing the likelihood of improper postures, risky workarounds, and ultimately WMSDs. These findings indicate the importance of addressing not just physical hazards but also the psychosocial aspects of desludging work. Chronic musculoskeletal issues can cripple the sanitation workforce, leading to staffing shortages and potential public health risks. Furthermore, WMSDs translate to increased healthcare burdens and decreased worker productivity. Our findings align with similar studies conducted in diverse contexts such as Zimbabwe [66], Denmark [67], and Norway [68] which likewise elucidated a connection between perceived effort and WMSDs. Therefore, interventions aimed at improving work ergonomics, providing mental health support, and fostering a more positive work environment could be crucial for reducing WMSDs and promoting a healthy desludging workforce.

Ultimately, this evidence generated supports the necessity for sanitation workers in Uganda to receive ergonomic training and education regarding WMSDs prevention and mitigation. Such training should cover preventive measures particular to the ergonomic dangers of the various body parts [47]. It is thus recommended that the above preventive measures are adopted and operationalized by the leadership of the desludging operators and other respective stakeholders such as local government health and safety regulatory bodies, non-governmental organizations (NGOs) focused on worker health and safety, professional associations representing sanitation workers, and healthcare providers specializing in occupational health.

## Conclusion and recommendations

In conclusion, WMSDs are prevalent among desludging operators in Uganda. Desludging operators’ ability to influence the availability of equipment needed to do their work and frequency of feeling that everything done was an effort were significantly associated with WMSDs. Interventions should focus on ensuring adequate provision of ergonomic equipment and promoting practices that reduce the physical strain associated with desludging tasks. Additionally, comprehensive training programs addressing proper lifting techniques and posture awareness could significantly mitigate the risk of WMSDs among desludging operators, thus safeguarding their health and well-being while enhancing productivity in the desludging sector.

### Limitations of the study

While this study adds to the knowledge base on WMSDs, it also has some limitations. The study relied on self-reported data through the NMQ, which is susceptible to recall bias and cannot definitively diagnose specific conditions. Also, this study recognizes limitations related to measurement of key ergonomic factors. Additionally, the cross-sectional design precludes causal inferences between identified factors and WMSDs. Furthermore, the potential for vague responses or exaggeration of symptoms by participants and the relatively small sample size might have limited the study’s generalizability and the strength of identified associations. Future research could consider adopting more rigorous designs, such as larger prospective cohorts, to strengthen the evidence base on WMSDs among desludging operators in Uganda.

## Data Availability

The datasets used and/or analysed during the current study are available from the corresponding author on reasonable request.

## Abbreviations

FSTP: Fecal Sludge Treatment Plant
GKMA: Greater Kampala Metropolitan Area
ILO: International Labour Organisation
MGLSD: Ministry Of Gender Labour And Social Development
MSDs: Musculoskeletal Disorders
NGOs: Non-Governmental Organizations
NMQ: Nordic Musculoskeletal Questionnaire
OSH: Occupational Safety And Health
WHO: World Health Organization
WMSDs: Work-Related Musculoskeletal Disorders
WWTP: Wastewater Treatment Plant
YLD: Years Lived With Disability
